# Do continuous forms of intra-operative ultrafiltration enhance recovery after adult cardiac surgery with cardiopulmonary bypass? A protocol for systematic review and meta-analysis of randomized controlled trials

**DOI:** 10.1186/s13643-021-01826-y

**Published:** 2021-10-08

**Authors:** Joel Bierer, David Horne, Roger Stanzel, Mark Henderson, Leah Boulos, Jill Hayden

**Affiliations:** 1 Division of Cardiac Surgery, Department of Surgery, Dalhousie University, Halifax, Canada; 2 Department of Clinical Perfusion, Nova Scotia Health Authority, Halifax, Canada; 3 Maritime SPOR SUPPORT Unit, Halifax, Canada; 4 Department of Community Health & Epidemiology, Dalhousie University, Halifax, Canada

**Keywords:** Continuous, Ultrafiltration, Adult, Cardiac surgery, Cardiopulmonary bypass

## Abstract

**Background:**

Cardiac surgery with cardiopulmonary bypass (CPB) is associated with a systemic inflammatory syndrome that adversely impacts cardiopulmonary function and can contribute to prolonged postoperative recovery. Intra-operative ultrafiltration during CPB is a strategy developed by pediatric cardiac specialists, aiming to dampen the inflammatory syndrome by removing circulating cytokines and improving coagulation profiles during the cardiac operation. Although ultrafiltration is commonly used in the pediatric population, it is not routinely used in the adult population. This study aims to evaluate if randomized evidence supports the use of continuous intra-operative ultrafiltration to enhance recovery for adults undergoing cardiac surgery with CPB.

**Methods:**

This systematic review and meta-analysis will include randomized controlled trials (RCT) that feature continuous forms of ultrafiltration during adult cardiac surgery with CPB, specifically assessing for benefit in mortality rates, invasive ventilation time and intensive care unit length of stay (ICU LOS). Relevant RCTs will be retrieved from databases, including MEDLINE, Embase, CENTRAL and Scopus, by a pre-defined search strategy. Search results will be screened for inclusion and exclusion criteria by two independent persons with consensus. Selected RCTs will have study demographics and outcome data extracted by two independent persons and transferred into RevMan. Risk of bias will be independently assessed by the Revised Cochrane Risk-of-Bias (RoB2) tool and studies rated as low-, some-, or high- risk of bias. Meta-analyses will compare the intervention of continuous ultrafiltration against comparators in terms of mortality, ventilation time, ICU LOS, and renal failure. Heterogeneity will be measured by the *χ*^2^ test and described by the *I*^2^ statistic. A sensitivity analysis will be completed by excluding included studies judged to have a high risk of bias. Summary of findings and certainty of the evidence, determined by the GRADE approach, will display the analysis findings.

**Discussion:**

The findings of this systematic review and meta-analysis will summarize the evidence to date of continuous forms of ultrafiltration in adult cardiac surgery with CPB, to both inform adult cardiac specialists about this technique and identify critical questions for future research in this subject area.

**Systematic review registration:**

This systematic review and meta-analysis is registered in PROSPERO CRD42020219309 (https://www.crd.york.ac.uk/prospero/display_record.php?ID=CRD42020219309).

**Supplementary Information:**

The online version contains supplementary material available at 10.1186/s13643-021-01826-y.

## Background

Cardiac surgery utilizing cardiopulmonary bypass (CPB) produces an inflammatory syndrome due to blood passing through an artificial extracorporeal circuit [1]. The primary mechanism is thought to be complement activation through the alternate pathway, which culminates in cytokine activation, leukocyte extravasation and, ultimately, tissue edema, and dysfunction [1, 2]. Clinically, this systemic inflammatory reaction can feature postoperative myocardial suppression, parenchymal lung injury, and systemic vasodilation, which can prolong intensive care needs and delay recovery [3].

In the early 1990s, pediatric cardiac surgeons developed intra-operative ultrafiltration to dampen this inflammatory syndrome associated with cardiopulmonary bypass [4]. This technique featured a membrane with pores that allow fluid and substances less than 65 kDa, including a broad profile of inflammatory mediators, to be removed from the patient's circulation. Multiple ultrafiltration types were developed, which can be characterized by being continuous or non-continuous. Continuous forms of ultrafiltration are utilized throughout the entire cardiopulmonary bypass time and include zero-balance ultrafiltration (ZBUF), dilutional ultrafiltration (DUF), and subzero ultrafiltration (SBUF). Non-continuous forms of ultrafiltration are utilized at the end of CPB or after completion of CPB, including conventional ultrafiltration (CUF), modified ultrafiltration (MUF), and simplified modified ultrafiltration (SMUF). Various techniques can also be combined, for example, ZBUF-MUF. In the pediatric literature, there is some evidence that high-intensity continuous forms of ultrafiltration can reduce ventilation time and intensive care length of stay, while non-continuous forms such as MUF reduce bleeding complications and transfusion requirements [4].

In the late 1990s, adult cardiac surgeons also began to examine the benefits of intra-operative ultrafiltration. A systematic review and meta-analysis of non-continuous forms of ultrafiltration in adult cardiac surgery concluded there is a significant reduction in bleeding complications, which matches the pediatric literature [5]. However, there has not been a systematic review of the clinical benefits of all high-intensity continuous forms of ultrafiltration, despite there being suggestions in the pediatric literature of reducing ventilation time and intensive care unit length of stay. This review aims to assess the current level of evidence, through randomized control trials, for continuous ultrafiltration forms on improving postoperative clinical outcomes for adults undergoing cardiac surgery.

## Methods

The design and protocol of this systematic review and meta-analysis are derived from the Cochrane Handbook’s guidelines for Systematic Reviews of Interventions and reports using the Preferred Reporting Items for Systematic Reviews and Meta-analyses Protocols (PRISMA-P) [6, 7]. Please see Additional file 1 for a completed PRISMA-P checklist. This protocol is registered in the PROSPERO database under the identifier CRD42020219309.

### Data sources

An information specialist (LB) developed the search strategy in MEDLINE (Ovid MEDLINE All) and subsequently translated it to Embase (Elsevier), the Cochrane Central Registry of Controlled Trials (CENTRAL) (Wiley), and Scopus (Elsevier). The search strategy was designed to capture as much relevant literature as possible, including search terms related to continuous and non-continuous forms of ultrafiltration. Key search terms in the title, abstract, and keywords were as follows: cardiopulmonary bypass, ultrafiltration, zero-balance, dilutional, subzero, and conventional. The full search strategy in MEDLINE is provided in Additional file 2. Registered RCTs in progress will be captured by the search of CENTRAL, which encompasses major trials registers such as ClinicalTrials.gov. The reference lists of all included studies will be screened for additional relevant resources and a forward search for records citing the included studies will be executed using Scopus.

### Study types

Only randomized controlled trials (RCTs) published in English or another language will be assessed for inclusion. All other study designs will be excluded.

### Participants

The participants will be men or women of age greater than 18 undergoing cardiac surgery requiring cardiopulmonary bypass. The type of adult cardiac surgery includes, but is not limited to, coronary bypass surgery, valvular surgery, concomitant coronary-valve surgery, and aortic surgery; no type of adult cardiac surgery will be excluded. Patients under 18 years of age undergoing congenital heart surgery will be excluded.

### Intervention

The intervention will be any type of continuous intra-operative ultrafiltration therapy used through the CPB time. This includes conventional ultrafiltration (CUF), zero-balance ultrafiltration (ZBUF), dilutional ultrafiltration (DUF), and subzero ultrafiltration (SBUF) as well as combination techniques such as ZBUF with MUF (ZBUF-MUF). These techniques can be considered sufficiently similar, in terms of clinical outcomes of interests, for the purposes of this analysis. Non-continuous forms of ultrafiltration, such as MUF alone, will be excluded.

### Comparators

The comparator will be a non-intervention control, such as CPB without ultrafiltration or a comparison to another type of non-continuous ultrafiltration. MUF is used for a short time after the patient is weaned from CPB and is therefore considered non-continuous. CUF used only during the rewarming portion of CPB is also considered a non-continuous form of ultrafiltration as it is not utilized throughout the entire CPB time. We consider continuous and non-continuous forms of ultrafiltration to be sufficiently different, in terms of physiological and related clinical outcomes of interest for this analysis.

### Outcomes

The primary outcome will be operative mortality (death during the same hospitalization as cardiac operation or within 30 days of operation). Secondary outcomes will be invasive ventilation time (hours), intensive care unit length of stay (ICU LOS) (hours), the incidence of renal failure, stroke, sternal wound infection, pneumonia, bleeding complications, and patient-reported outcomes of recovery.

### Study selection

JB and DH will independently screen the titles and abstracts identified by searches, for possible study inclusion, through Covidence [8]. A third party (RS) will arbitrate any disagreement. Full-length publications of candidate studies will be acquired. JB and DH will independently screen the full texts to identify the RCTs that meet inclusion criteria; the Cochrane Study Collection form will be completed and is included in Additional file 3. We will record reasons for exclusion of the study. A third party (RS) will arbitrate any disagreement. This process will be documented using a PRISMA flow-chart [7].

### Data extraction

JB and DH will independently extract data from eligible studies by hand using the Cochrane Study Collection Form (see Additional file 3). Data will include: authors, publication date, randomization method, blinding, patient demographics (sex and mean age), surgical risk (low risk defined by STS or EuroScore II mortality risk score < 4 and moderate or high risk defined by STS or EuroScore II mortality risk score > 4), type of cardiac surgery (coronary bypass surgery, valvular surgery, concomitant coronary-valve surgery and aortic surgery), CPB time, aortic cross-clamp time, type of ultrafiltration in the intervention group, the group size of intervention and control arms, primary and secondary outcomes, and follow-up period (in-hospital follow-up or 30-day follow-up). Dichotomous variables will be extracted as the number and total group size. Continuous outcomes such as ventilation time and ICU LOS will all be converted to hours if reported in days. Any continuous variable that is expressed as mean and confidence interval will be converted to mean and standard deviation. A third party (RS) will arbitrate any disagreement. JB will directly transfer outcome data from accepted studies into RevMan [9]. Included studies that are missing specific outcome data will be recorded and included in the risk of bias assessment.

### Risk of bias

JB and DH will independently assess the risk of bias of included studies by completing the Revised Cochrane Risk-of-Bias (RoB2) tool [10]. A third party (RS) will arbitrate any disagreement. This process examines possible bias from randomization, deviation from intended intervention, measurement of outcome, missing outcome data, and the selection of reported results. Each included study will be rated as low-risk of bias, some concerns of bias, and high-risk of bias.

### Data synthesis

Data analysis will be done using RevMan and a forest plot for each outcome generated [9]. A random-effects model will be used because of the suspected heterogeneity in types of continuous ultrafiltration methods used; for example, ZBUF and ZBUF-MUF would both meet inclusion criteria. Furthermore, there could be heterogeneity in the underlying cardiac pathology and patient risk profile. If the data is not appropriate for a meta-analysis, a narrative synthesis will be completed in its place. Analysis of dichotomous outcomes will utilize the Peto odds ratio method, as the primary outcome of operative mortality and secondary outcomes such as renal failure, stroke, and severe bleeding are expected to be relatively rare [6]. Analysis of continuous outcomes will utilize mean difference as outcomes such as ventilation time, and ICU LOS will be recorded in the same unit of hours [6]. A meta-analysis will only be performed if there are at least two included studies reporting the same outcome.

Statistical heterogeneity will be measured by the *χ*^2^ test (*p* < 0.1) and described by the *I*^2^ statistic. *I*^2^ > 75% indicates significant heterogeneity, and this result will require investigation. Significant heterogeneity will be explored and described as clinical diversity (the type of cardiac surgery, risk profile of patients and type of ultrafiltration used), methodological diversity (outcome definition, outcome reporting and risk of bias in study design), and statistical diversity. If ten or more studies report on an outcome, reporting bias will be examined by completing a funnel plot analysis.

There will be one subgroup analysis that will differentiate patients by operative risk profile: low risk (STS or EuroScore II mortality risk score < 4) vs moderate or high risk (STS or EuroScore II mortality risk score > 4). Moderate and high-risk subgroups might also include patients with evidence of preoperative organ dysfunction, including preoperative renal, cardiopulmonary, and hepatic failure. Subgroup analysis will not be performed if fewer than two included studies report outcomes specific to the defined risk categories. Form test for subgroup interactions will be completed using RevMan [9].

Meta-analysis results will be interpreted in terms of both clinical and statistical significance. Any statistically significant reduction in operative mortality will be considered clinically significant. Any statistically significant reduction in renal failure, stroke, pneumonia, or sternal wound infection will be considered clinically significant as it has clearly shown that any one of these post-cardiac surgery complications are associated with a 15% reduction in survival at one year when compared to patients that had no complications [11]. The continuous outcomes of ventilation time and ICU LOS are often influenced by many clinical variables and are prolonged in the case of postoperative complications. Prolonged ventilation time, and therefore ICU LOS, over 24 h is an independent risk factor for mortality at 1 year [11]. Therefore, any statistically significant reduction in these outcomes will be of some clinical significance. To ensure clarity of any statistically significant effect size, both the % reduction and corresponding value in hours will be reported for ventilation time and ICU LOS.

### Sensitivity analysis

A sensitivity analysis will be completed to evaluate the meta-analysis results. Studies that are judged to be high-risk of bias, via the aforementioned Cochrane Risk of Bias Tool, will be excluded from the pooled analysis. These results will be examined and compared to the total pooled results. The meta-analysis results would be considered robust if the sensitivity analysis does not significantly change the meta-analysis findings.

### Quality of evidence

The quality of included evidence will be characterized, independently by JB and DH, through the Grading of Recommendations Assessment, Development and Evaluation (GRADE) [12]. Domains that determine the certainty of result through GRADE include risk of bias, the inconsistency of outcome results, indirectness of results, imprecision of results, suspicion of publication bias, effect size, plausible confounding, and dose-response gradient [12]. For each outcome, the table will list the effect of the intervention, effect of control, effect size, the difference between intervention and comparator, number of participants, number of studies included in outcome analysis, GRADE certainty of evidence, and a comment on interpretation [12]. GRADEpro software will be used to summarize this information in a Summary of Findings table [13].

## Discussion

This systematic review and meta-analysis seeks to gauge the current level of evidence of continuous forms of ultrafiltration, commonly used in pediatric cardiac surgery but uncommonly in adult cardiac surgery, for knowledge translation between the two patient populations. The review’s findings will yield information applicable to regular clinical practice and translational research initiatives to enhance recovery for adult patients undergoing cardiac surgery.

## Supplementary Information

**Additional file 1.** PRISMA-P 2015 Checklist.

**Additional file 2.** Full search strategy in MEDLINE.

**Additional file 3.** Cochrane Study Collection form.

## Data Availability

All data and materials will be readily available to interpret and replicate the findings of this article.

## Abbreviations

CPB: Cardiopulmonary bypass
CUF: Conventional ultrafiltration
DUF: Dilutional ultrafiltration
ICU: Intensive care unit
LOS: Length of stay
MUF: Modified ultrafiltration
SBUF: Subzero ultrafiltration
SMUF: Simplified modified ultrafiltration
STS: Society of Thoracic Surgeons
ZBUF: Zero-balance ultrafiltration
