# Supplementary material for: Do continuous forms of intra-operative ultrafiltration enhance recovery after adult cardiac surgery with cardiopulmonary bypass? A protocol for systematic review and meta-analysis of randomized controlled trials
Source: Syst Rev. 2021 Oct 8;10:265. doi: 10.1186/s13643-021-01826-y (PMC8499556; doi:10.1186/s13643-021-01826-y)
Supplement: Supplementary file 2 — Additional file 2. Full search strategy in MEDLINE. [file 13643_2021_1826_MOESM2_ESM.pdf]

Database: MEDLINE

1	Cardiopulmonary Bypass/
2	(cardiopulmonary bypass* or cardio pulmonary bypass* or cp bypass* or cpb).ti,ab,kw,kf.
3	heart lung bypass*.ti,ab,kw,kf.
4	lung heart bypass*.ti,ab,kw,kf.
5	or/1-4
6	Ultrafiltration/
7	Hemofiltration/
8	6 or 7
9	(continuous or dilutional or subzero or "sub zero" or zero or balance?).ti.
10	8 and 9
11	((ultrafiltration or h?emofiltration or filtration or purification) adj2 (continuous or dilutional or subzero or "sub zero" or zero or balance?)).ti,ab,kw,kf.
12	(duf or sbuf or s buf or zbuf or z buf).ti,ab,kw,kf.
13	or/10-12
14	5 and 13
15	(non continuous or noncontinuous or conventional or modified or simplified or combined).ti.
16	8 and 15
17	((ultrafiltration or h?emofiltration or filtration or purification) adj2 (non continuous or noncontinuous or conventional or modified or simplified or combined)).ti,ab,kw,kf.
18	(cuf or muf or smuf).ti,ab,kw,kf.
19	or/16-18
20	5 and 19
21	14 or 20
