# Supplementary material for: Do continuous forms of intra-operative ultrafiltration enhance recovery after adult cardiac surgery with cardiopulmonary bypass? A protocol for systematic review and meta-analysis of randomized controlled trials
Source: Syst Rev. 2021 Oct 8;10:265. doi: 10.1186/s13643-021-01826-y (PMC8499556; doi:10.1186/s13643-021-01826-y)
Supplement: Supplementary file 3 — Additional file 3. Cochrane Study Collection form. [file 13643_2021_1826_MOESM3_ESM.pdf]

Cochrane

Data collection form

Intervention review – RCTs only

No single form will be appropriate for all reviews. This form should be used as a starting point for to consider the information you need to collect for your review, and design your own form accordingly. Sections can be expanded and added, and irrelevant sections should be removed. Information included should be comprehensive, for use in the text of your review, 'Characteristics of included studies' table, risk of bias assessment, and statistical analysis.

Notes on using a data collection form:

- Record any missing information as unclear or not described, to make it clear that the information was not found in the study report(s), not that you forgot to extract it.
- Include any instructions and decision rules on the data collection form, or in an accompanying document. It is important to practice using the form and give training to any other authors using the form.
- The form fields in the document allow the form to be completed while locked for editing, or can be deleted.

**Notes:**

General Information

**Study ID** *(e.g. author name, year)*	
**Form completed by**	
**Notes:**	

Characteristics of included studies

Methods

	**Descriptions as stated in report/paper**	**Location in text or source** *(pg & ¶/fig/table/other)*
**Aim of study** *(e.g. efficacy, equivalence, pragmatic)*		
**Design** *(e.g. parallel, crossover, cluster)*		
**Start & end dates**		
**Total study duration**		
**Study funding sources** *(including role of funders)*		
**Possible conflicts of interest** *(for study authors)*		
**Notes:**		

Participants

	Description<br>*Include comparative information for each intervention or comparison group if available*		Location in text or source *(pg & ¶/fig/table/other)*
**Setting**			
**Type of Cardiac Surgery**			
**Cardiac Surgery Risk (high, medium, low)**			
**Inclusion criteria**			
**Exclusion criteria**			
**Total no. randomized**			
**Clusters** *(if applicable, no., type, no. people per cluster)*			
**No. randomized per group** *(specify whether no. people or clusters)*	*Group 1 name*	*Group 2 name*	
**No. missing** *(if overall, e.g. exclusions & withdrawals, whether or not missing from analysis)*			
**Reasons missing**			
**No. missing** *(if by group, e.g. exclusions & withdrawals, whether or not missing from analysis)*			
**Reasons missing**			
**No. participants moved from one group to another**			
**Reasons moved**			
**Baseline imbalances**			
**Age**			
**Sex (proportion male)**			
**Cardiopulmonary Bypass Time (min)**			
**Aortic Cross Clamp Time (min)**			
**Race/Ethnicity**			
**Subgroups measured** *(e.g. split by age or sex)*			
**Subgroups reported**			

Intervention groups

Intervention Group 1

	Description as stated in report/paper	Location in text or source *(pg & ¶/fig/table/other)*
**Group name** *(from paper or abbreviation)*		
**Theoretical basis and type of UF** *(include key references)*		
**Description** *(include sufficient detail for replication, e.g. components)*		
**Duration of treatment period**		
**Co-interventions**		
**Economic information** *(e.g. pedometer cost, changes in other costs as result of intervention)*		
**Integrity of delivery**		
**Compliance**		
**Total Ultrafiltrate Volume**		
**Notes:**		

Comparator Group 1

	Description as stated in report/paper	Location in text or source *(pg & ¶/fig/table/other)*
**Group name** *(from paper or abbreviation)*		
**Theoretical basis and type of UF** *(include key references)*		
**Description** *(include sufficient detail for replication, e.g. components)*		
**Duration of treatment period**		
**Co-interventions**		
**Economic information** *(e.g. pedometer cost, changes in other costs as result of intervention)*		

**Integrity of delivery**		
**Compliance**		
**Total Ultrafiltrate Volume**		
**Notes:**		

Data and analysis

Dichotomous outcome – Operative Mortality (30 Days)

	Description as stated in report/paper				Location in text or source *(pg & ¶/fig/table/other)*
**Outcome definition** *(with diagnostic criteria if relevant)*					
**Imputation of missing data** *(e.g. assumptions made for ITT analysis)*					
**Power** *(e.g. power & sample size calculation, level of power achieved)*					
**Results**	**Intervention**		**Comparison**		
	No. with event	No. measured	No. with event	No. measured	
(time point or subgroup, copy rows as needed)					
**Time points measured but not reported**					
**Any other results reported** *(e.g. odds ratio, risk difference, CI or P value)*					
**Statistical methods used and appropriateness of these** *(e.g. adjustment for correlation)*					
**Reanalysis required?** *(specify)*	☐ Yes ☐ No ☐ Unclear				
**Reanalysis possible?**	☐ Yes ☐ No ☐ Unclear				
**Reanalyzed results**					
**Notes:**					

Continuous outcome – ICU Length of Stay (hours)

	**Description as stated in report/paper**	**Location in text or source** *(pg & ¶/fig/table/other)*
**Outcome definition** *(with diagnostic criteria if relevant)*		
**Unit of measurement** *(if relevant)*		
**Scales: upper and lower limits** *(indicate whether high or low score is good)*		
**Is outcome/tool validated?**	☐ Yes ☐ No ☐ Unclear	
**Minimally important difference**		
**Imputation of missing data** *(e.g. assumptions made for ITT analysis)*		
**Power** *(e.g. power & sample size calculation, level of power achieved)*		

**Results**	**Intervention**			**Comparison**			
	Mean	SD *(or other variance, specify)*	No. participants	Mean	SD *(or other variance, specify)*	No. participants	
(time point or subgroup, copy rows as needed)							

**Time points measured but not reported**		
**Any other results reported** *(e.g. mean difference, CI, P value)*		
**Statistical methods used and appropriateness of these** *(e.g. adjustment for correlation)*		
**Reanalysis required?** *(specify)*	☐ Yes ☐ No ☐ Unclear	
**Reanalysis possible?**	☐ Yes ☐ No ☐ Unclear	
**Reanalyzed results**		

Notes:

Continuous outcome – Ventilation Time (hours)

	Description as stated in report/paper	Location in text or source *(pg & ¶/fig/table/other)*
**Outcome definition** *(with diagnostic criteria if relevant)*		
**Person measuring/reporting**		
**Unit of measurement** *(if relevant)*		
**Scales: upper and lower limits** *(indicate whether high or low score is good)*		
**Is outcome/tool validated?**	☐ Yes ☐ No ☐ Unclear	
**Minimally important difference**		
**Imputation of missing data** *(e.g. assumptions made for ITT analysis)*		
**Power** *(e.g. power & sample size calculation, level of power achieved)*		
**Post-intervention or change from baseline?**		

**Results**	**Intervention**			**Comparison**			
	Mean	SD *(or other variance, specify)*	No. participants	Mean	SD *(or other variance, specify)*	No. participants	
(time point or subgroup, copy rows as needed)							

**Time points measured but not reported**		
**Any other results reported** *(e.g. mean difference, CI, P value)*		
**Statistical methods used and appropriateness of these** *(e.g. adjustment for correlation)*		
**Reanalysis required?** *(specify)*	☐ Yes ☐ No ☐ Unclear	
**Reanalysis possible?**	☐ Yes ☐ No ☐ Unclear	
**Reanalyzed results**		
**Notes:**		

Dichotomous outcome – Acute Kidney Injury, Failure, Dialysis

	Description as stated in report/paper				Location in text or source *(pg & ¶/fig/table/other)*
**Outcome definition** *(with diagnostic criteria if relevant)*					
**Imputation of missing data** *(e.g. assumptions made for ITT analysis)*					
**Power** *(e.g. power & sample size calculation, level of power achieved)*					
**Results**	**Intervention**		**Comparison**		
	No. with event	No. measured	No. with event	No. measured	
(time point or subgroup, copy rows as needed)					
**Time points measured but not reported**					
**Any other results reported** *(e.g. odds ratio, risk difference, CI or P value)*					
**Statistical methods used and appropriateness of these** *(e.g. adjustment for correlation)*					
**Reanalysis required?** *(specify)*	☐ Yes ☐ No ☐ Unclear				
**Reanalysis possible?**	☐ Yes ☐ No ☐ Unclear				
**Reanalyzed results**					
**Notes:**					

Dichotomous outcome – Stroke

	Description as stated in report/paper				Location in text or source *(pg & ¶/fig/table/other)*
**Outcome definition** *(with diagnostic criteria if relevant)*					
**Imputation of missing data** *(e.g. assumptions made for ITT analysis)*					
**Power** *(e.g. power & sample size calculation, level of power achieved)*					
**Results**	**Intervention**		**Comparison**		

	No. with event	No. measured	No. with event	No. measured	
(time point or subgroup, copy rows as needed)					
**Time points measured but not reported**					
**Any other results reported** *(e.g. odds ratio, risk difference, CI or P value)*					
**Statistical methods used and appropriateness of these** *(e.g. adjustment for correlation)*					
**Reanalysis required?** *(specify)*	☐ Yes ☐ No ☐ Unclear				
**Reanalysis possible?**	☐ Yes ☐ No ☐ Unclear				
**Reanalyzed results**					
**Notes:**					

Dichotomous outcome – Sternal Wound Infection

	**Description as stated in report/paper**				**Location in text or source** *(pg & ¶/fig/table/other)*
**Outcome definition** *(with diagnostic criteria if relevant)*					
**Imputation of missing data** *(e.g. assumptions made for ITT analysis)*					
**Power** *(e.g. power & sample size calculation, level of power achieved)*					
**Results**	**Intervention**		**Comparison**		
	No. with event	No. measured	No. with event	No. measured	
(time point or subgroup, copy rows as needed)					
**Time points measured but not reported**					
**Any other results reported** *(e.g. odds ratio, risk difference, CI or P value)*					
**Statistical methods used and appropriateness of these** *(e.g. adjustment for correlation)*					

**Reanalysis required?** *(specify)*	☐ Yes ☐ No ☐ Unclear		
**Reanalysis possible?**	☐ Yes ☐ No ☐ Unclear		
**Reanalyzed results**			
**Notes:**			

Dichotomous outcome – Bleeding Complication req Transfusion

	Description as stated in report/paper				Location in text or source *(pg & ¶/fig/table/other)*
**Outcome definition** *(with diagnostic criteria if relevant)*					
**Imputation of missing data** *(e.g. assumptions made for ITT analysis)*					
**Power** *(e.g. power & sample size calculation, level of power achieved)*					
**Results**	**Intervention**		**Comparison**		
	No. with event	No. measured	No. with event	No. measured	
(time point or subgroup, copy rows as needed)					
**Time points measured but not reported**					
**Any other results reported** *(e.g. odds ratio, risk difference, CI or P value)*					
**Statistical methods used and appropriateness of these** *(e.g. adjustment for correlation)*					
**Reanalysis required?** *(specify)*	☐ Yes ☐ No ☐ Unclear				
**Reanalysis possible?**	☐ Yes ☐ No ☐ Unclear				
**Reanalyzed results**					
**Notes:**					

Dichotomous outcome – Pneumonia

	Description as stated in report/paper				Location in text or source *(pg & ¶/fig/table/other)*
**Outcome definition** *(with diagnostic criteria if relevant)*					
**Imputation of missing data** *(e.g. assumptions made for ITT analysis)*					
**Power** *(e.g. power & sample size calculation, level of power achieved)*					
**Results**	**Intervention**		**Comparison**		
	No. with event	No. measured	No. with event	No. measured	
(time point or subgroup, copy rows as needed)					
**Time points measured but not reported**					
**Any other results reported** *(e.g. odds ratio, risk difference, CI or P value)*					
**Statistical methods used and appropriateness of these** *(e.g. adjustment for correlation)*					
**Reanalysis required?** *(specify)*	☐ Yes ☐ No ☐ Unclear				
**Reanalysis possible?**	☐ Yes ☐ No ☐ Unclear				
**Reanalyzed results**					
**Notes:**					

Other outcome type – Patient Reported Outcomes on Recovery

	Description as stated in report/paper	Location in text or source *(pg & ¶/fig/table/other)*
**Outcome definition** *(with diagnostic criteria if relevant)*		
**Person measuring/reporting**		
**Unit of measurement** *(if relevant)*		
**Scales: upper and lower limits** *(indicate whether high or low score is good)*		

**Is outcome/tool validated?**	☐ Yes ☐ No ☐ Unclear				
**Imputation of missing data** *(e.g. assumptions made for ITT analysis)*					
**Power** *(e.g. power & sample size calculation, level of power achieved)*					
**Results**	Intervention result	SE (or other variance)	Control result	SE (or other variance)	
(time point or subgroup, copy rows as needed)					
	Overall results		SE (or other variance)		
(time point or subgroup, copy rows as needed)					
**Time points measured but not reported**					
**Any other results reported** *(e.g. mean difference, CI, P value)*					
**Unit of analysis** *(by individuals, cluster/groups or body parts)*					
**Statistical methods used and appropriateness of these** *(e.g. adjustment for correlation)*					
**Reanalysis required?** *(specify)*	☐ Yes ☐ No ☐ Unclear				
**Reanalysis possible?**	☐ Yes ☐ No ☐ Unclear				
**Reanalyzed results**					

Dichotomous outcome – Other

	**Description as stated in report/paper**				**Location in text or source** *(pg & ¶/fig/table/other)*
**Outcome definition** *(with diagnostic criteria if relevant)*					
**Imputation of missing data** *(e.g. assumptions made for ITT analysis)*					
**Power** *(e.g. power & sample size calculation, level of power achieved)*					
**Results**	**Intervention**		**Comparison**		
	No. with event	No. measured	No. with event	No. measured	

(time point or subgroup, copy rows as needed)					
**Time points measured but not reported**					
**Any other results reported** *(e.g. odds ratio, risk difference, CI or P value)*					
**Statistical methods used and appropriateness of these** *(e.g. adjustment for correlation)*					
**Reanalysis required?** *(specify)*	☐ Yes ☐ No ☐ Unclear				
**Reanalysis possible?**	☐ Yes ☐ No ☐ Unclear				
**Reanalyzed results**					
**Notes:**					

Continuous outcome – Other

	Description as stated in report/paper		Location in text or source *(pg & ¶/fig/table/other)*
**Outcome definition** *(with diagnostic criteria if relevant)*			
**Unit of measurement** *(if relevant)*			
**Scales: upper and lower limits** *(indicate whether high or low score is good)*			
**Is outcome/tool validated?**	☐ Yes ☐ No ☐ Unclear		
**Minimally important difference**			
**Imputation of missing data** *(e.g. assumptions made for ITT analysis)*			
**Power** *(e.g. power & sample size calculation, level of power achieved)*			

**Results**	**Intervention**			**Comparison**			
	Mean	SD *(or other variance, specify)*	No. participants	Mean	SD *(or other variance, specify)*	No. participants	
(time point or subgroup, copy rows as needed)							

**Time points measured but not reported**			
**Any other results reported** *(e.g. mean difference, CI, P value)*			
**Statistical methods used and appropriateness of these** *(e.g. adjustment for correlation)*			
**Reanalysis required?** *(specify)*	☐ Yes ☐ No ☐ Unclear		
**Reanalysis possible?**	☐ Yes ☐ No ☐ Unclear		
**Reanalyzed results**			
**Notes:**			

Other information

	Description as stated in report/paper	Location in text or source *(pg & ¶/fig/table/other)*
**Key conclusions of study authors**		
**References to other relevant studies**		
**Correspondence required for further study information** *(from whom, what and when)*		
**Notes:**		

Definitions

Change from baseline	A measure for a continuous outcome calculated as the difference between the baseline score and the post-intervention score.
Clusters	A group of participants who have been allocated to the same intervention arm together, as in a cluster-randomized trial, e.g. a whole family, town, school or patients in a clinic may be allocated to the same intervention rather than separately allocating each individual to different arms.
Co-morbidities	The presence of one or more diseases or conditions other than those of primary interest. In a study looking at treatment for one disease or condition, some of the individuals may have other diseases or conditions that could affect their outcomes.
Compliance	Participant behavior that abides by the recommendations of a doctor, other health care provider or study investigator (also called adherence or concordance).
Exclusions	Participants who were excluded from the study or the analysis by the investigators.
Imputation	Assuming a value for a measure where the true value is not available (e.g. assuming last observation carried forward for missing participants).
Integrity of delivery	The degree to which the specified procedures or components of an intervention are delivered as originally planned.
Post-intervention	The value of an outcome measured at some time point following the beginning of the intervention (may be during or after the intervention period).
Power	In clinical trials, power is the probability that a trial will obtain a statistically significant result when the true intervention effect is a specified size. For a given size of effect, studies with more participants have greater power. Note that power should not be considered in the risk of bias assessment.
Providers	The person or people responsible for delivering an intervention and related care, who may or may not require specific qualifications (e.g. doctors, physiotherapists) or training.
Reanalysis	Additional analysis of a study's results by a review author (e.g. to introduce adjustment for correlation that was not done by the study authors).
Sociodemographic	Social and demographic information about a study or its participants, including economic and cultural information, location, age, gender, ethnicity, etc.
Theoretical basis	The use of a particular theory (such as theories of human behavior change) to design the components and implementation of an intervention
Unit of allocation	The unit allocated to an intervention arm. In most studies individual participants will be allocated, but in others it may be individual body parts (e.g. different teeth or joints may be allocated separately) or clusters of multiple people.
Unit of analysis	The unit used to calculate N in an analysis, and for which the result is reported. This may be the number of individual people, or the number of body parts or clusters of people in the study.
Unit of measurement	The unit in which an outcome is measured, e.g. height may be measured in cm or inches; depression may be measured using points on a particular scale.
Validation	A process to test and establish that a particular measurement tool or scale is a good measure of that outcome.

Sources:

Cochrane Collaboration Glossary, 2010. Available from http://www.cochrane.org/training/cochrane-handbook.

Higgins JPT, Green S (editors). Cochrane Handbook for Systematic Reviews of Interventions Version 5.1.0 [updated March 2011]. The Cochrane Collaboration, 2011. Available from http://community-archive.cochrane.org/glossary.

Last JM (editor), A Dictionary of Epidemiology, 4th Ed. New York: Oxford University Press, 2001.
